# *In vivo* dual-delivery of glucagon like peptide-1 (GLP-1) and dipeptidyl peptidase-4 (DPP4) inhibitor through composites prepared by microfluidics for diabetes therapy

**DOI:** 10.1039/c6nr00294c

**Published:** 2016-04-20

**Authors:** F. Araújo, N. Shrestha, M. J. Gomes, B. Herranz-Blanco, D. Liu, J. J. Hirvonen, P. L. Granja, H. A. Santos, B. Sarmento

**Affiliations:** a I3S – Instituto de Investigação e Inovação em Saúde , Universidade do Porto , 4200-135 Porto , Portugal . Email: bruno.sarmento@ineb.up.pt ; Tel: +351 220408800; b INEB – Instituto de Engenharia Biomédica , Universidade do Porto , 4200-135 Porto , Portugal; c ICBAS – Instituto Ciências Biomédicas Abel Salazar , Universidade do Porto , 4150-180 Porto , Portugal; d Division of Pharmaceutical Chemistry and Technology , Faculty of Pharmacy , University of Helsinki , FI-00790 Helsinki , Finland; e CESPU , Instituto de Investigação e Formação Avançada em Ciências e Tecnologias da Saúde , 4585-116 Gandra , Portugal

## Abstract

Oral delivery of proteins is still a challenge in the pharmaceutical field. Nanoparticles are among the most promising carrier systems for the oral delivery of proteins by increasing their oral bioavailability. However, most of the existent data regarding nanosystems for oral protein delivery is from *in vitro* studies, lacking *in vivo* experiments to evaluate the efficacy of these systems. Herein, a multifunctional composite system, tailored by droplet microfluidics, was used for dual delivery of glucagon like peptide-1 (GLP-1) and dipeptidyl peptidase-4 inhibitor (iDPP4) *in vivo*. Oral delivery of GLP-1 with nano- or micro-systems has been studied before, but the simultaneous nanodelivery of GLP-1 with iDPP4 is a novel strategy presented here. The type 2 diabetes mellitus (T2DM) rat model, induced through the combined administration of streptozotocin and nicotinamide, a non-obese model of T2DM, was used. The combination of both drugs resulted in an increase in the hypoglycemic effects in a sustained, but prolonged manner, where the iDPP4 improved the therapeutic efficacy of GLP-1. Four hours after the oral administration of the system, blood glucose levels were decreased by 44%, and were constant for another 4 h, representing half of the glucose area under the curve when compared to the control. An enhancement of the plasmatic insulin levels was also observed 6 h after the oral administration of the dual-drug composite system and, although no statistically significant differences existed, the amount of pancreatic insulin was also higher. These are promising results for the oral delivery of GLP-1 to be pursued further in a chronic diabetic model study.

## Abstract 2

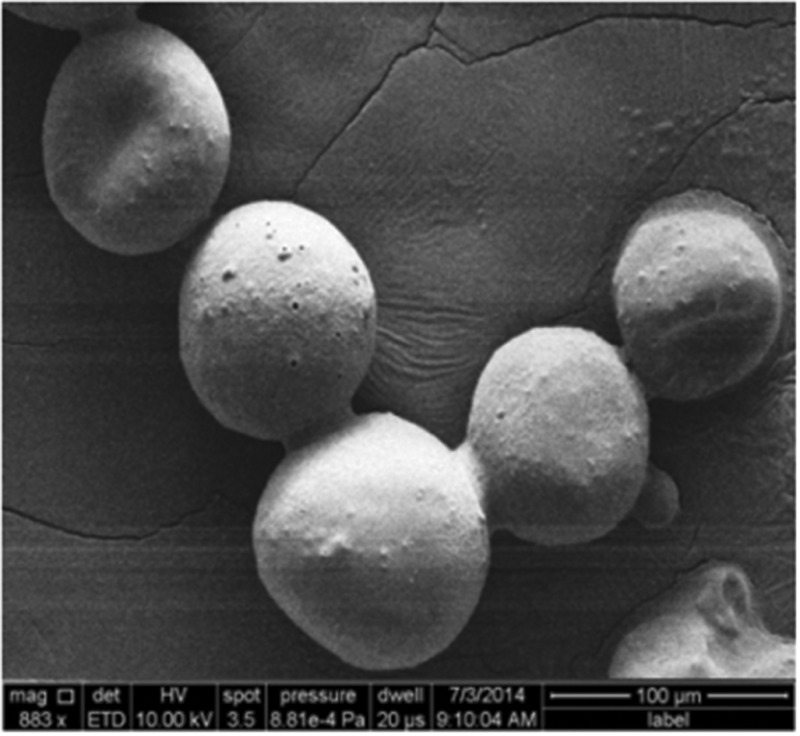
Nanoparticles are promising carrier systems for the oral delivery of proteins, a challenge in the pharmaceutical field.

## Introduction

The development of therapeutic proteins has increased exponentially over the past few decades.^1^ Owing to their natural origin, proteins and peptides have exquisite effectiveness, activity, specificity and relatively lower toxicity, which has a significant impact in the treatment of numerous diseases.^2^ However, due to their delicate structure, they must be administrated through parenteral routes. The use of proteins as therapeutic agents for oral delivery is still underachieved, which keeps the desired oral administration of proteins yet in its infancy.^3,4^

With the expanding knowledge in nanomedicine the development of nanosized delivery systems is revolutionizing the pharmaceutical field, by considerably improving the therapeutic effects of most of the drugs and their bioavailability. Nanoparticles are also regarded as promising nanocarriers for oral protein delivery.^5,6^ These nanoparticles are formed by biomaterials that can be tailored towards the desired administration route and can further be associated with other molecules in order to improve their interaction with the target organ.^7^ Nevertheless, despite the huge efforts in the development of nanoparticles, most of the existent data essentially addresses *in vitro* studies, lacking *in vivo* experiments to evaluate the efficacy of the developed nanosystems.

Herein, an innovative approach for type 2 diabetes mellitus (T2DM) therapy is proposed. Within the multifunctional tailorable composite system for dual-drug delivery previously described,^8^ glucagon-like peptide-1 (GLP-1) and a dipeptidyl peptidase-4 (DPP4) inhibitor (iDPP4) drug were combined in a single system, aiming to overcome the side effects associated with each one of them separately. Due to its incretin effect (insulin secretion after a meal, in a glucose-dependent manner) GLP-1 is one of the most promising therapeutic molecules for T2DM therapy, avoiding the well-known hypoglycemic effects of current drugs.^9,10^ Nevertheless, GLP-1 has a very short half-life, being cleaved by the DPP4 enzyme in less than 2 minutes.^11^ This system was assembled through the droplet microfluidics technique^8^ and it is based on the use of polymeric poly(lactic-*co*-glycolic acid) (PLGA) nanoparticles. These nanoparticles were claimed as adequate candidates to provide a protective, stable and biocompatible environment to the encapsulated peptides and proteins.^2,12,13^ The nanoparticles were functionalized with chitosan (CS) and a cell-penetrating peptide (CPP) and, as previously described by our group, showed high and strong interactions with intestinal cells *in vitro*.^8,14^ CS is a positively charged polymer used to a large extent due to its advantageous characteristics of adhesion to negatively charged mucosae and cell membranes, thereby increasing cell permeability of intestinal cells by transiently opening tight junctions and affecting the paracellular and intracellular pathways, without changing the junctional morphology or causing any damage to the cells.^15,16^ In turn, CPP has the ability to cross the cellular membranes without causing a cellular response, increasing the transcellular transport.^17,18^ We have previously shown *in vitro* that the dual-release of the peptide and the drug had a synergistic effect regarding GLP-1 permeability. In the presence of the iDPP4, the activity of the enzyme responsible for degrading GLP-1, thus considerably decreasing its bioavailability, was drastically reduced, further improving the amount of active peptide permeated *in vitro* across the intestinal cell monolayers.^8^ Additionally, using the microfluidics system, the functionalized nanoparticles were encapsulated within an enteric polymer – hydroxypropylmethylcellulose acetylsuccinate (HPMC-AS) – conferring pH-sensitivity to the system and thus enabling the release of nanoparticles only under the simulated intestinal conditions.^19,20^ This synergetic effect was also described for other carriers.^21,22^ Both PLGA and HPMC-AS polymers are approved by the Food and Drug Administration (FDA) for parenteral and food administration, respectively. Previous studies also show that they are extensively used in the drug delivery field without toxic effects after acute and chronic administrations.^8,13,23,24^

Pursuing these promising results, in the present work the developed system was orally delivered to T2DM rat model, induced by the combination of streptozotocin (STZ) and nicotinamide drugs. Blood glucose, plasmatic insulin levels, and insulin pancreatic content were quantified over time during this study.

## Experimental

### Materials

GLP-1 acetate (7–37, MW 3355.7 Da) was purchased from United Peptides (USA). iDPP4 (NVP DPP 728 dihydrochloride, MW 375.77 Da) was purchased from Tocris Bioscience (UK) and CPP R9 was purchased from GenicBio (China). PLGA 50 : 50 was obtained from Corbion Purac, Purasorb® PDLG 5004A, The Netherlands. Polyvinyl alcohol (PVA), medium molecular weight CS, 2-(*N*-morpholino)-ethanesulfonic acid (MES), 1-ethyl-3-(3-dimethylaminopropyl)-carbodiimide (EDC), *N*-hydroxysuccinimide (NHS), STZ, nicotinamide and isoflurane were purchased from Sigma-Aldrich (USA). HPMC-AS was obtained from Shin-Etsu (Japan). Pluronic® F127 was purchased from BASF (Germany). Lancet Unistik 2 Normal Fixed Depth Lancet Needle 2.4 mm 21 Gauge was purchased from Owen Mumford, Ltd (France) and FreeStyle Precision Blood Glucose Test Strips from Abbott Diabetes Care (Portugal).

### Preparation of GLP-1 loaded PLGA–CS nanoparticles

PLGA nanoparticles were produced based on the water-in-oil-in-water (w/o/w) double emulsion technique, through the modified solvent emulsification–evaporation method, using 2% of PVA as a surfactant, as described elsewhere.^8,25^ Afterwards, in order to functionalize the nanoparticles with CS, the formulation was added into a CS solution at a ratio of 1 : 2 (w/w), regarding the solid content of the solutions, and left overnight under magnetic stirring.^8^

### CPP conjugation to the CS-functionalized nanoparticles

EDC/NHS coupling chemistry was used to covalently conjugate the free amine groups in the CS structure with the carboxylic group of CPP, as previously described.^8^ Briefly, the CS-functionalized nanoparticles were dispersed in MES solution containing EDC and NHS (pH 5.5.). CPP was then added to this dispersion in a ratio of 1 : 10 (CPP : nanoparticles, w/w) and the conjugation occurred overnight in the dark under 300 rpm stirring, forming PLGA + CS–CPP.

### Characterization of nanoparticles

The nanoparticles were characterized with respect to their average size (*Z*-average), polydispersity (PdI) and surface charge zeta ((ζ)-potential) by dynamic light scattering using a Malvern Zetasizer Nano ZS instrument (Malvern Instruments Ltd, UK).

The association efficiency (AE) and loading degree (LD) of GLP-1 were calculated by the difference between the total amount of GLP-1 used to prepare the nanoparticles and the amount of GLP-1 that remained in the aqueous phase after the nanoparticle isolation by centrifugation at 20 000*g* for 30 min at 4 °C. The amount of GLP-1 was determined by high performance liquid chromatography (HPLC), as described elsewhere.^8^

### Microfluidics enteric encapsulation of nanoparticles

The GLP-1 loaded nanoparticles were encapsulated within the HPMC-AS pH-sensitive polymer, loaded with the iDPP4, using a double emulsion technique through a microfluidic flow-focusing glass device. The preparation of the co-drug loaded multifunctional systems was previously described in detail.^8^ The modified nanoparticles encapsulated in the HPMC-AS polymer are defined as H-PLGA particles.

The shape, size, morphology and surface topography of the enteric encapsulated particles were assessed by scanning electron microscopy (SEM, Zeiss DSM 962, Germany). The AE of the iDPP4 was calculated by dissolving the enteric encapsulated particles in a pH 7.4 solution. The amount of iDPP4 was determined by HPLC, as described elsewhere.^8^

### Type 2 diabetic animals

Male Wistar rats, 7-weeks old, weighing 150–200 grams, obtained from Harlan Laboratories, Inc. (Spain) were used for the study. They were maintained under standard laboratory conditions (12 h light/dark cycles, temperature of 21 ± 2 °C and relative humidity of 35% to 60%). They were fed with standard pellets and water *ad libitum*.

The rats were randomly divided into 6 groups, with 5 animals per group. The groups were named according to the different formulations that were given as oral gavage: group 1 – normal animals (control group of normal rats and with no T2DM induction); group 2 – phthalate buffer solution at pH 4.0 (control group); group 3 – GLP-1 and iDPP4 aqueous solution; group 4 – H-PLGA empty particles; group 5 – H-PLGA particles loaded with GLP-1 (H-PLGA-GLP-1); and group 6 – H-PLGA particles loaded with GLP-1 and iDPP4 (H-PLGA-GLP-1-iDPP4) (study group). The amount of administered particles was equivalent to a GLP-1 content of 200 μg per kg of weight of the rat. This dose was chosen taking into account the administration doses of the GLP-1 analogs that are in the market [liraglutide (Victoza®) and exenatide (Byetta®)], which is around 20–30 μg kg^–1^ and a previous study performed by Huotari and co-workers where 50 μg of GLP-1 was s.c. administrated per mouse.^26^

Animal experiments were approved by the Local Ethics Committee at the University of Porto and conducted under the guidelines and recommendations of FELASA and the European Directive 2010/63/EU.

Induction of T2DM in animals, with the exception of group 1, was done in overnight-fasted rats, by an intraperitoneal (i.p.) injection of 120 mg per kg of nicotinamide and, 15 min later, 60 mg per kg of STZ. STZ was freshly dissolved in citrate buffer (0.1 M; pH, 4.5) and nicotinamide was dissolved in normal physiological saline buffer (pH 7.4), maintained on ice prior to use.^27^

Glucose tolerance was determined by the intraperitoneal glucose tolerance test (IPGTT) 3 days after the T2DM induction. Overnight-fasted animals were administered i.p. with a glucose solution (2 g kg^–1^). Blood samples were taken by puncturing with the help of a lancet from the tail tip at different time points (–15, 30, 60, 90, and 120 min) after glucose administration. Blood glucose was measured using a glucometer Precision Xtra (Abbott Diabetes Care, Portugal) by placing a small drop of blood on a new test strip and recording the measurements.

### Hypoglycemic effect *in vivo*

After proving the successful T2DM induction through the IPGTT experiments, different formulations were orally administrated in a phthalate buffer (pH 4.0), according to the groups defined in the previous section and blood glucose and insulin levels in plasma and pancreas were measured.

#### Blood glucose measurements

The blood samples were withdrawn from the tail vein and blood glucose levels were measured for 8 h at different time points (0, 0.5, 1, 2, 4, 6 and 8 h) after administration. The area under the curve (AUC) over 8 h was calculated for each group. The total hypoglycemic decrease (HD%) in serum glucose levels was calculated as follows.^3,28^
1
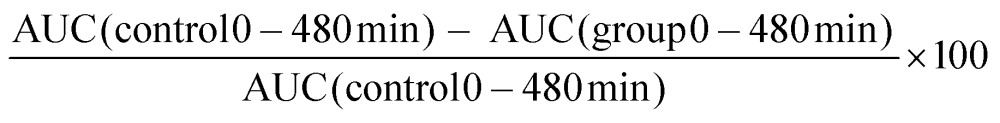



#### Plasmatic insulin measurements

At the time points of 0, 2 and 6 h after administration, the withdrawn blood was collected into Eppendorf tubes containing 0.5 M of EDTA (10% of the final volume) to prevent blood clotting. The samples were centrifuged at 5400*g* for 10 min at 4 °C. The supernatants were collected and stored at –20 °C until further studies. Insulin quantification was done according to the manufacturer's instructions using a rat insulin ELISA kit from Mercodia (Sweden).

#### Pancreatic insulin content determination

At the end of the study, the animals were sacrificed by cervical dislocation, after isoflurane anesthesia, and the pancreases were extracted. The pancreases were isolated by removing the fat and the connective tissues, and were weighed. Then, they were placed separately in 3 mL of ice cold acid ethanol (0.18 M HCl in 70% ethanol) and kept on ice. The pancreases were further homogenized with a probe sonicator and 3 mL more of acid ethanol were added into the tube of the tissue homogenate and stored overnight at 4 °C. Afterwards, the samples were centrifuged at 3500 rpm for 45 min at 4 °C and the supernatant was transferred to another tube to be stored at –20 °C. These steps were repeated twice more and the supernatants from the different centrifugations were collected together. Before the quantification of the insulin content, using the rat insulin ELISA kit from Mercodia (Sweden), samples were left at room temperature, mixed by vortexing, and diluted 1000–1500 times.

### Statistical analysis

All the experiments were performed in triplicate and represented as mean ± standard deviation (SD). A Student *t*-test and one-way analysis of variance (ANOVA) with unpaired and Bonferroni post-test (GraphPadPrism, GraphPad software Inc., CA, USA) were used to analyze the data, respectively. The level of significance was set at probabilities of *p < 0.05, **p < 0.01, and ***p < 0.001.

## Results and discussion

### Characterization of nanoparticles

As previously demonstrated by our group, CS is able to increase the permeability of the antidiabetic peptide GLP-1 across the intestinal cells.^25,29^ Due to its positive surface charge, CS conferred a positive charge to the nanoparticles, which increases the interaction with the negatively charged intestinal cells and mucus layer. Additionally, CPP was also conjugated to the CS-functionalized PLGA nanoparticles, further enhancing the intestinal permeability of the GLP-1-loaded CS-modified nanoparticles.^8,14,30,31^

**Fig. 1:**
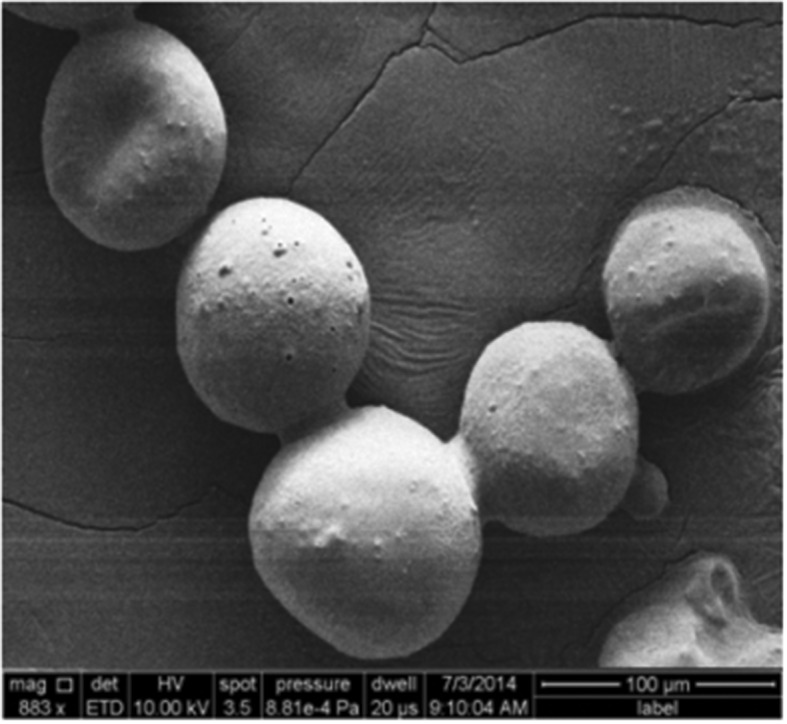
SEM image of CS-CPP modified nanoparticles encapsulated in the HPMC-AS polymer (H-PLGA particles).

After production and CS + CPP surface functionalization, the PLGA nanoparticles were characterized on the basis of the different physicochemical parameters that are known to have an impact on their interaction with cells and on the drug delivery.^32^ The mean size, PdI, surface charge (ζ-potential), AE and LD of GLP-1 were evaluated, as shown in Fig. 1. Comparing the non-modified nanoparticles with the CS + CPP conjugated nanoparticles, an increase in size from 174 ± 5 to 351 ± 4 nm, an increase in PdI and an inverse of charge from –20 ± 2 to 40 ± 0 mV was observed, proving efficient modification of the prepared PLGA nanoparticles.^8,25^ Due to the addition of CS + CPP to the system, the final mass was increased resulting in the decrease of the LD from 0.017 ± 0.03 to 0.08 ± 0.01. However, despite these surface modifications, the AE did not significantly change (approximately 70%) (Table 1).

**Table 1:** Characterization of the nanoparticles with respect to their size, PdI, ζ-potential, AE and LD of GLP-1. Results are presented as mean ± SD (*n* ≥ 3)

	PLGA	PLGA + CS	PLGA + CS + CPP
Size (nm)	174.4 ± 4.9	286.7 ± 5.5	351.3 ± 3.5
PdI	0.120 ± 0.045	0.188 ± 0.015	0.210 ± 0.024
ζ-potential (mV)	–20.0 ± 1.5	34.7 ± 2.8	40.0 ± 0.1
AE (%)	69.5 ± 10.3	∼69.5 ± 10.3	∼69.5 ± 10.3
LD (%)	0.017 ± 0.030	0.008 ± 0.001	0.008 ± 0.001

Further encapsulation of the CS + CPP modified nanoparticles with a pH-sensitive HPMC-AS polymer was made using the droplet microfluidics technique,^19,20^ to form H-PLGA particles. These particles presented sizes around 60 ± 7 μm with a regular and smooth surface and a spherical shape (Fig. 1). The similarities between the particles are characteristic of the microfluidic production technique, which originates uniform structures, with the additional advantage of providing an AE of nearly 100%.^8,19,20^ The AE of iDPP4 was 21 ± 4%. Since HPMC-AS is only soluble at pH 6.0 or higher, under low pH conditions of the gastric milieu, the particles will remain intact protecting the integrity of the CS + CPP modified nanoparticles.^8,20^

### *In vivo* assessment of the antidiabetic effect

A non-obese T2DM rat model induced by the administration of STZ combined with nicotinamide, was firstly proposed by Masiello and co-workers in 1998.^27^ This model was described as being the most suitable to study the biochemical and pharmacological antidiabetic drug effects, and was previously used to test GLP-1 analogs.^16,33,34^ It is based on the protective effects of nicotinamide against the β-cytotoxic effects caused by STZ, a widely used drug to induce diabetes mellitus in rodents.^35^ This model presents a number of features similar to T2DM, as described in detail elsewhere.^34^

Briefly, it induces stable and moderate hyperglycemia, does not require exogenous insulin for animals to survive, results in glucose intolerance, reduction of β cells and the presence (although impaired) of glucose-stimulated insulin secretion.^36^

**Fig. 2:**
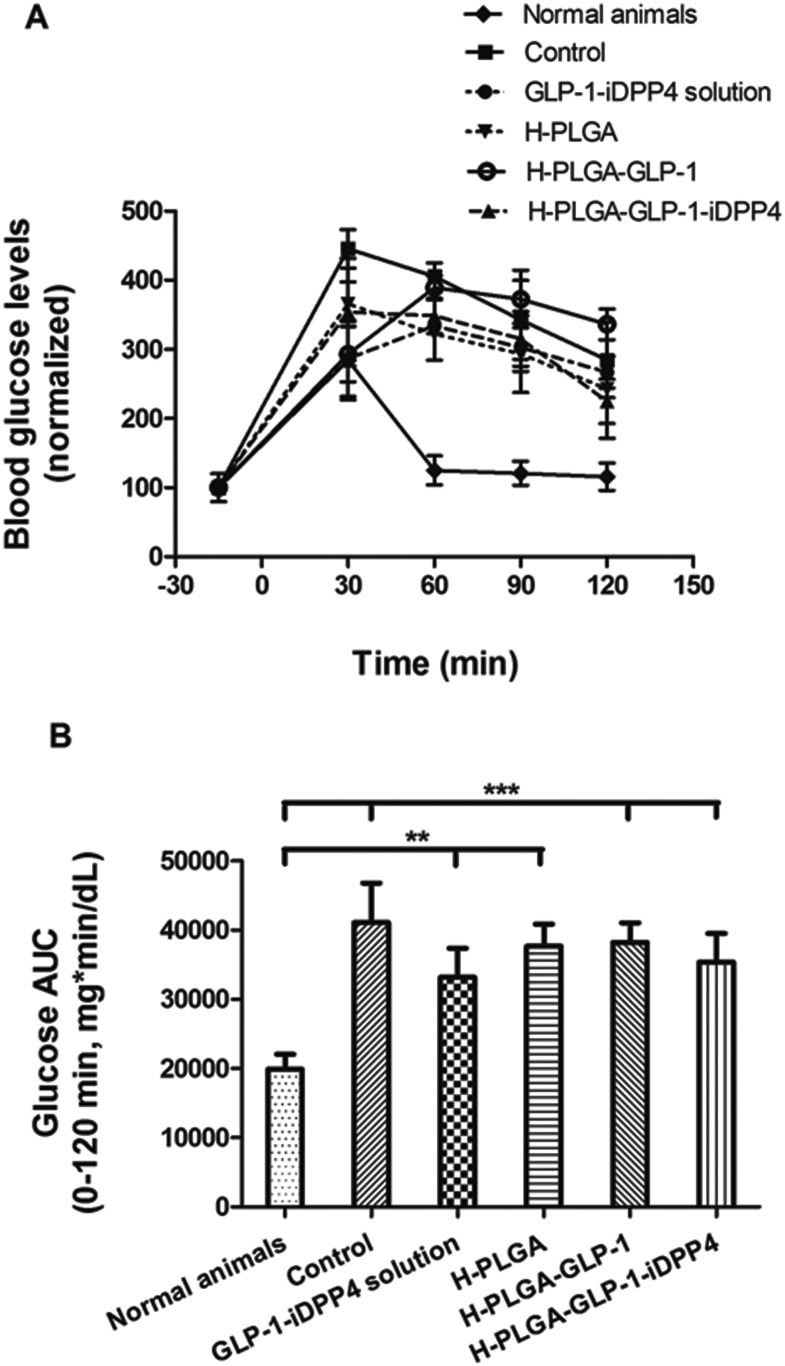
Blood glucose levels of T2DM-induced rats following i.p. administration of a glucose solution (2 g kg^–1^). The values were normalized by the normal animals’ group. Results are presented as mean ± SD (*n* = 5) (A). Blood glucose AUC in the period of –15–120 min after glucose administration. Results are presented as mean ± SD (*n* = 5). The levels of significance were set at probabilities of **p < 0.01, and ***p < 0.001, as compared with the normal animals’ group (B).

After 3 days of STZ–nicotinamide administration, a glucose tolerance test (IPGTT) was performed to verify the efficacy of the induction of T2DM.^34^ The glucose tolerance test was used to assess the ability of the body to metabolize glucose, and thus, to detect disorders in glucose metabolism. After the i.p. administration of glucose, the blood glucose levels were measured for 2 h in the different groups. As shown in Fig. 2A, the normal animals’ group had a rapid increase in the blood glucose levels 30 min after glucose administration, but the levels reached normal values 60 min after glucose administration and were constant, thereafter, *i.e.*, the animals were tolerant to glucose.^37^ In contrast, the other groups could not recover from the glucose administration. There was a maximum peak between 30 and 60 min, similar to the normal animals, but the blood glucose levels never reached the normal values in the 2 h period after glucose injection. As shown in Fig. 2B, all the groups presented a higher and statistically significant AUC compared to the normal animals’ group, in the period of –15–120 min of the study, which indicates that all the animals were intolerant to glucose, and thus, were considered as presenting T2DM.^37^

**Fig. 3:**
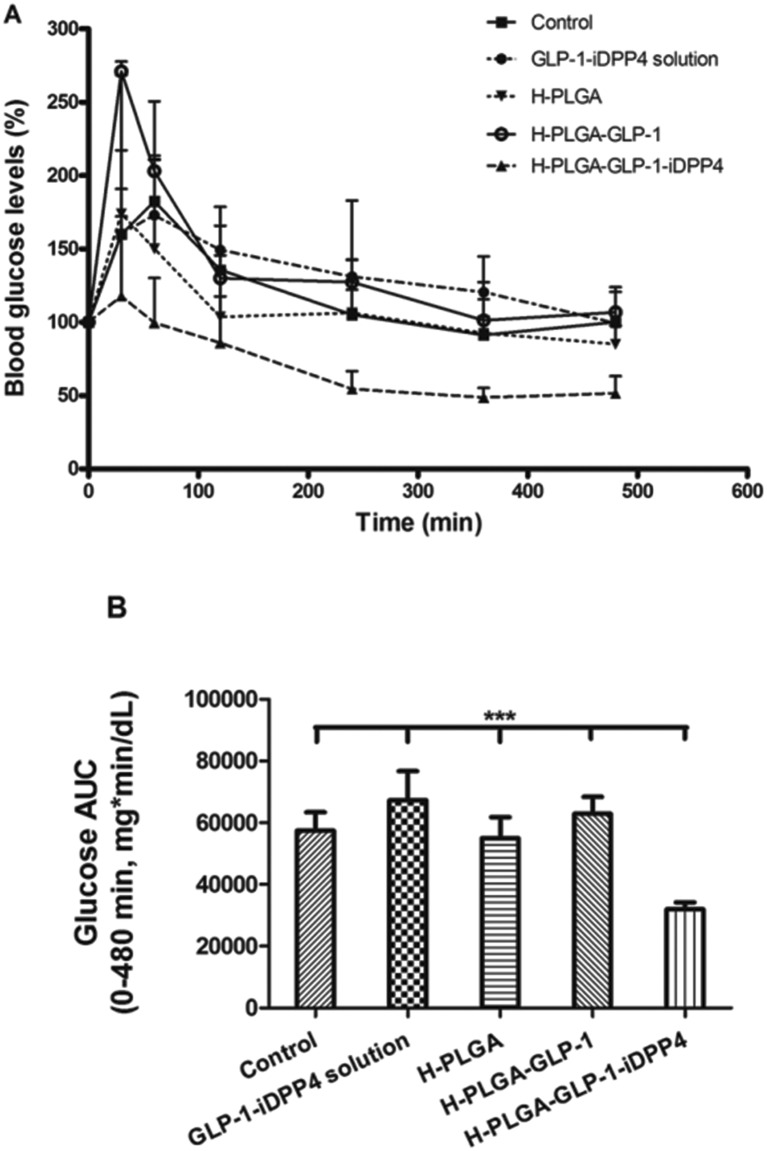
Blood glucose levels of T2DM-induced rats following oral administration of phthalate buffer solution (control), GLP-1-iDPP4 solution, H-PLGA particles, H-PLGA-GLP-1 particles and H-PLGA-GLP-1-iDPP4 particles. Results are presented as mean ± SD (*n* = 5) (A). Blood glucose AUC in the period of 0–480 min after oral administration. Results are presented as mean ± SD (*n* = 5). The levels of significance were set at the probability of ***p < 0.001, as compared with the H-PLGA-GLP-1-iDPP4 (B).

To evaluate the efficacy of our developed composite system, different formulations were orally administrated to the animals through gavage, as previously described in the Experimental section. The drug-loaded H-PLGA particles (H-PLGA-GLP-1-iDPP4) showed remarkable hypoglycemic effects (decrease in the blood glucose levels) in a sustained and prolonged manner. 2 h after administration, the blood glucose levels decreased significantly, remaining low until the end of the experiment (8 h). In comparison with other groups, at 4, 6 (*p* < 0.05) and 8 h (*p* < 0.001) after administration, results with H-PLGA-GLP-1-iDDP4 particles were statistically different (Fig. 3A). The determination of glucose AUC of the whole experiment (0–480 min), shown in Fig. 3B, proved that the GLP-1 and iDPP4 co-loaded particles had a statistically significant effect in decreasing the overall glucose levels compared to the control and the pure drugs, as well as in comparison with the empty H-PLGA and H-PLGA-GLP-1 particles (*p* < 0.01). The hypoglycemic decrease (HD%) presented in Table 2 also gives consistency to the obtained results, showing a HD% of approximately 0 for the GLP-1-iDPP solution and H-PLGA-GLP-1, 4.3 ± 6.4% for the empty H-PLGA particles, which is a negligible hypoglycemic efficacy,^28^ and 44.3 ± 4.0% for the DPP4-loaded H-PLGA particles. This hypoglycemic efficacy was similar^28^ or even higher^38,39^ than the results obtained in other studies where GLP-1 analogs were tested. Since the analogs are resistant to the DPP4 enzyme activity, we can assume that the dual delivery of GLP-1 and iDPP4 was effective, and that they had a synergetic effect regarding the blood glucose levels, as previously described for the *in vitro* experiments.^8,24^ Moreover, the functionalization of the PLGA nanoparticles improved the permeability of cells to GLP-1 and contributed to the overall increase in efficacy.^8,25^

**Table 2:** Total hypoglycemic decrease (HD%) in serum glucose levels in the 8 h experiment, with regard to the control

GLP-1-iDPP4 solution	H-PLGA	H-PLGA-GLP-1	H-PLGA-GLP-1-iDPP4
∼0	4.3 ± 3.6	∼0	44.3 ± 2.9

Being a powerful insulinotropic peptide, GLP-1 stimulates pancreatic insulin secretion and release in a glucose-dependent manner. Even in a T2DM scenario, the insulin response to GLP-1 remains intact,^40^ which means that after administration of GLP-1 in therapeutic dosages, the insulin secretory function can be restored in T2DM patients.^41^ This glucose dependency assures the safety of GLP-1 over other agents in the market that increase insulin secretion *via* glucose-independent mechanisms.^38,39^

**Fig. 4:**
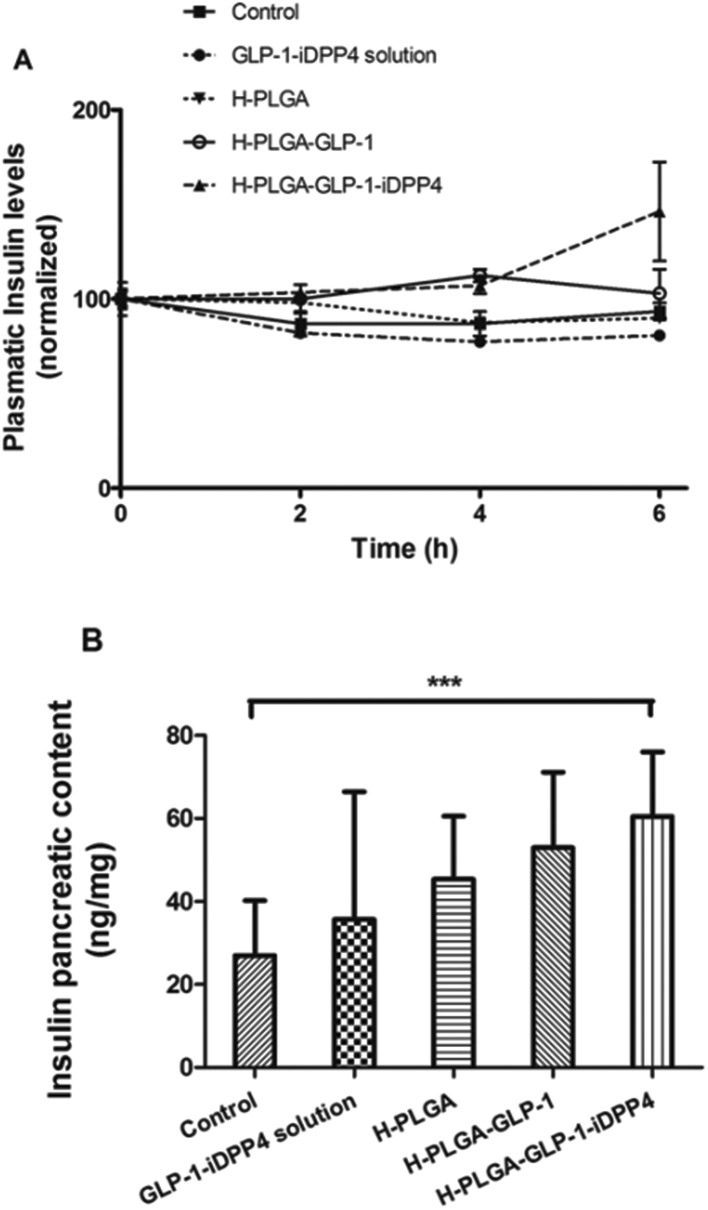
Plasmatic insulin levels in T2DM-induced rats following oral administration of buffer solution (control), GLP-1-iDPP4 solution, H-PLGA empty particles and H-PLGA-GLP-1-iDPP4 particles. Results are presented as mean ± SD (*n* = 5) (A). Pancreatic insulin content 8 h after oral administration of buffer solution (control), GLP-1-iDPP4 solution, H-PLGA empty and H-PLGA-GLP-1-iDPP4 particles. Data are shown as mean ± SD (*n* = 5). The level of significance was set at the probability of ***p < 0.001 between the control group and the H-PLGA-GLP-1-iDPP4 (B).

Thus, the plasmatic insulin levels were also measured for the time points of 0, 2 and 6 h after oral administration. As is depicted in Fig. 4A, no differences between the groups were found for the initial time points (0 and 2 h). However, at 6 h after oral administration, the H-PLGA-GLP-1-iDPP4 particles presented significantly higher plasmatic insulin levels compared to the control, the oral GLP-1-iDPP4 solution, the H-PLGA empty particles and the H-PLGA-GLP-1 groups (*p* < 0.001).

These results are in accordance with the blood glucose level measurements where the blood glucose levels were lower at the time point of 6 h (*p* < 0.05) in comparison with the 0 and 2 h time points. This is also in agreement with another study that showed an insulin increase in a slow, but prolonged manner over 8 h.^39^

The insulin pancreatic contents of the different groups after oral administration were also evaluated at the end of the 8 h experiment, as shown in Fig. 4B. The H-PLGA-GLP-1-iDPP4 group presented a higher amount of insulin compared with the other groups; however, this difference was only statistically significant with regard to the control group. These results might be due to the single dose administration, while an increase in the insulin pancreatic content was reported to exist only in long-term studies.^42–46^ Thus, although promising, these results must be followed up by a chronic diabetic model system.

Overall, the combined administration of GLP-1 and iDPP4 resulted in an increase in the hypoglycemic effects after oral administration, namely a decrease of blood glucose levels and enhancement of the insulin secretion. A clear improvement of the therapeutic efficacy of GLP-1 was observed in the presence of the iDPP4.^47–49^

## Conclusions

In this work, a dual-drug delivery multifunctional composite system was prepared through the highly reproducible microfluidics technique. The system was loaded with GLP-1 and iDPP-4 and tested *in vivo* in a non-obese type 2 diabetes mellitus rat model, induced by streptozotocin and nicotinamide. The combination of both GLP-1 and iDPP-4 resulted in an increase in the hypoglycemic effects in a sustained and prolonged manner. The glucose AUC was significantly lower than the control group, with a hypoglycemic decrease of 44%. An enhancement of the plasmatic insulin levels was also observed 6 h after the oral administration of the system. These are very promising results towards the development of oral protein/peptide delivery systems for T2DM therapy.
